# Wood Sponge for Oil–Water Separation

**DOI:** 10.3390/polym16162362

**Published:** 2024-08-21

**Authors:** Chang Zhang, Taoyang Cai, Shangjie Ge-Zhang, Pingxuan Mu, Yuwen Liu, Jingang Cui

**Affiliations:** 1 College of Science, Northeast Forestry University, Harbin 150040, China; zc2021@nefu.edu.cn (C.Z.);; 2 Aulin College, Northeast Forestry University, Harbin 150040, China

**Keywords:** wood sponge, sewage treatment, crude oil adsorption, hydrophobic, adsorption mechanism

## Abstract

In addition to filtering some sediments, hydrophobic wood sponges can also absorb many organic solvents, particularly crude oil. The leakage of crude oil poses a serious threat to the marine ecosystem, and oil mixed with water also generates great danger for its use. From the perspective of low cost and high performance, wood sponges exhibit great potential for dealing with crude oil pollution. Wood sponge is a renewable material. With a highly oriented layered structure and a highly compressible three-dimensional porous frame, wood sponges are extremely hydrophobic, making them ideal for oil–water separation. Currently, the most common approach for creating wood sponge is to first destroy the wood cell wall to obtain a porous-oriented layered structure and then enhance the oil–water separation ability via superhydrophobic treatment. Wood sponge prepared using various experimental methods and different natural woods exhibits distinctive properties in regards to robustness, compressibility, fatigue resistance, and oil absorption ability. As an aerogel material, wood sponge offers multi-action (absorption, filtration) and reusable oil–water separation functions. This paper introduces the advantages of the use of wood sponge for oil–water separation. The physical and chemical properties of wood sponge and its mechanism of adsorbing crude oil are explained. The synthesis method and the properties are discussed. Finally, the use of wood sponge is summarized and prospected.

## 1. Introduction

With the large-scale exploitation and use of oil, the technology regarding oil–water separation has been developing. A large number of water-driven crude oil production methods will inevitably mix water with oil (asphalt, resin, and naphthenate in oil act as emulsifiers, making it difficult for water to gather and separate) [1,2,3,4]. And the presence of this moisture will greatly affect the transportation and safe use of oil. In addition, the increase in oil spills also poses a great threat to the ecological environment and the safety of biological life. However, due to the increasing industrial demand, the scale of crude oil exploitation will only increase [3,5,6,7,8,9,10,11,12]. At present, the simplest and safest methods for oil–water separation include the use of hydrophobic and lipophilic oil-absorbing agents. However, most of the adsorption materials now include carbon-based aerogels such as activated carbon, graphene, and carbon nanotubes [13,14,15]. There are also bio-based aerogels such as chitosan aerogels [16], cellulose aerogels [17], and chitin aerogel [18]. In addition, there are artificial polymer aerogels such as commercial sponge, melamine, silicone sponge, etc., which have been employed in the field of oil–water separation [19,20,21]. However, due to the need for expensive equipment, its imperfect mechanical properties, and a complicated preparation process, its large-scale application is seriously hindered [22]. Other physical methods, such as centrifugal separation and air flotation [23], membrane separation [24,25,26], gravity separation [27,28], photoelectrocatalysis [29], and other schemes, offer poor separation selectivity, low reliability, and high cost [30,31,32]. Chemical methods such as the use of dispersants, combustion [33], and biodegradation not only exhibit difficulty in regards to crude oil recovery, but also cause secondary pollution [7,34,35,36,37,38,39,40] and other problems. In the study of the separation of water and organic solvents, the biomass-based adsorbent in the form of carbon aerogel has shown the ability to realize both a light weight and the recyclability of organic solvents, achieving good results [37,38,39,40]. However, the mixed water solution, with crude oil as an organic solvent, requires sufficient robustness of the adsorption materials in order to achieve success.

As a new adsorption material, wood sponge is a lightweight, degradable, and highly compressible 3D aerogel porous material [41,42,43]. At the same time, because the main skeleton structure is cellulose, and natural wood is rich in cellulose, it is also rich in raw materials, low in cost, and environmentally friendly. The structure of wood is directional and hierarchical. After the cell wall of the wood cells is chemically treated to remove lignin and hemicellulose, it will leave behind a hard cellulose skeleton, with good stability [44,45,46,47,48]. At this point, wood is basically composed of cellulose microfibers, which is a cellulose aerogel, to a certain extent, with a specific solvent adsorption capacity and extremely high porosity, with holes are closely arranged along the longitudinal direction of the wood fibers to form a series of relatively uniform low-curvature pipes [49,50,51,52]. Because the spring-like layered structure endows the wood sponge with sufficient mechanical compressibility and elasticity, it can withstand repeated extrusion without incurring structural damage. This type of wood displays a number of sponge-like characteristics, so it is called wood sponge.

**Figure 1:**
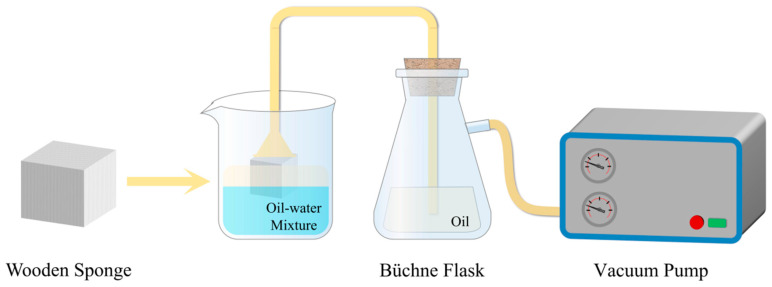
A hydrophobic wood sponge collects crude oil using a vacuum pump.

It is obvious that wood sponge exhibits great potential for absorbing organic solvents, and the appearance of wood sponge is only the beginning of its applications in sewage treatment for the adsorption of crude oil. However, wood sponge displays natural hydrophilicity and lipophilicity. If it is to be used for oil–water separation and the absorption of oil or organic solvent, it must be modified to be hydrophobic. Generally, the chemical deposition method is used to modify wood sponge. After hydrophobic modification of wood sponge using a chemical modifier, the porous structure was still observed in the cross-sectional plane. Under the enlarged observation of a radial section, it was noted that the modifier was uniformly deposited on the large surface of the sample in the form of small particles [53,54,55]. In mainstream research articles about hydrophobic sponge, the water contact angle of the treated wood can reach levels great than 100, the density is generally only 50–60 mg cm^−3^, and the porosity ranges from at least 90% up to 98% [56]. And under the same strain, the stress required by modified wood sponge is less than that of unmodified wood sponge. This light, hydrophobic, and oleophilic wood sponge can not only filter sewage containing crude oil but also absorb crude oil directionally, which also shows that targeted hydrophobic modification treatment has little effect on the structure of wood sponge. Generally, the oil absorption method is employed to directly place the hydrophobic sponge into the oil–water mixture, or to connect a sealed collection device through one end of a conduit; the collection device is connected to a vacuum pump, and the other end is connected to a hydrophobic wood sponge and placed into the oil–water mixture to filter out the water from the crude oil and to introduce the pollutant into the absorber, and it is then absorbed in the direction of the fiber bundle [53,54,57,58]. The device for testing the oil absorption capacity using a vacuum pump is shown in Figure 1.

This paper points out the necessity of using wood sponge for oil–water separation (part I), and introduces the mechanism of absorbing crude oil originating from the characteristics of wood sponge (Section 2). The preparation methods for wood sponge are summarized, and different preparation methods are compared (Section 3). Next, the limitations of wood sponge used in oil–water separation are briefly introduced (Section 4), and the future of wood sponge used in oil–water separation is prospected (Section 5).

## 2. Sewage Treatment Mechanism of Adsorption of Crude Oil by Wood Sponge

### 2.1. Characteristics of Wood Sponge

Wood sponge, as a new material obtained by treating natural wood materials using chemical or physical methods, exhibits unique and diverse physical and chemical properties. The sewage treatment mechanism of wooden sponge for the adsorption of crude oil is mainly based on its unique material characteristics and structural advantages.

Thanks to its rich pore structure, the appearance of wood sponge is usually sponge-like or foam-like, offering excellent extrusion recovery and cutting ability, which can be adapted to the adsorption requirements of various shapes and surfaces, and it is not easily damaged during use [53]. Its density (40–80 kg m^−3^) [54,59] is much lower than that of many other types of sponges or foam materials, making wood sponges more convenient to store, transport, and use. Its high porosity of more than 80% also means that it contains many tiny pores and channels, which not only provide abundant storage space for liquids (such as water or oil), but also enhance the capillary adsorption capacity of wood sponges [53,60]. Generally speaking, the higher the porosity, the better the adsorption performance of wood sponge [61].

Moreover, the three-dimensional porous network structure provides the wood sponge with a large specific surface area, increasing its surface contact with liquid, thus improving the adsorption efficiency [62,63,64,65]. Its three-dimensional network structure also helps to maintain the mechanical stability and durability of the wood sponge.

In the preparation process, lignin, hemicellulose, and other components in natural wood materials are usually stripped using chemical reagents, and the cellulose skeleton is retained, thus forming the unique structure of wood sponge [53]. Because cellulose is a natural polymer compound, wood sponge possesses good biodegradability [59,66]. This characteristic endows the porous polymer with incomparable advantages: it will not cause long-term pollution to the environment, and it conforms to the concept of sustainable development [67].

In addition, the wood sponge shows good stability in general chemical environments because the easily reactive substances have been stripped in advance using chemical reagent pretreatment [61]. However, it should be noted that some strong acids, strong bases, or organic solvents may destroy the structure and properties of wood sponges [68].

Most importantly, wood sponge exhibits great modification potential and can meet the needs of additional fields. Through further chemical or physical treatment, wood sponge can be endowed with more functional characteristics. For example, hydrophobic wood sponge can be prepared by hydrophobic treatment to improve its oil absorption performance [53,57], or superelastic sponge is prepared via superelastic treatment to increase its elasticity and ease of use [69,70].

The main criteria for evaluating the excellent properties of hydrophobic wood sponge are: internal water contact angle, porosity and recyclability [20,71], oil absorption capacity [72,73], separation efficiency [57], and compressive elasticity [63], along with other parameters. Recyclability refers to the ability of hydrophobic wood sponge to repeatedly absorb and squeeze crude oil from sewage containing petroleum, which reflects the compression fatigue resistance and repetitive work ability of hydrophobic wood sponge, largely determining whether it can actually be used to treat crude oil sewage. Oil absorption capacity is the ratio of the oil absorption capacity of hydrophobic wood sponge to its own mass, which focuses on the hydrophobic and oleophilic capacity of wood sponge at this stage. The separation efficiency is similar to the oil absorption capacity, but it is the ratio of the quality difference in the oil–water mixture, before and after absorption, to that of the original crude oil quality. Compressive elasticity is the maximum compression height ratio of hydrophobic wood sponge perpendicular to the fiber direction, which can restore its original state after compression; this is also a prominent feature of high porosity and deformability. Although wood itself exhibits high porosity and resilience, with the deepening of theoretical research, the methods for preparing hydrophobic wood sponge are becoming more and more mature and advanced, and the robustness, compression fatigue, hydrophobicity, and oil absorption ability of wood sponge are increasing. Table 1 shows the results of separating the oil–water mixture using wood sponge and other materials.

### 2.2. Mechanism of Crude Oil Adsorption

The mechanism of adsorption of crude oil by wood sponge is mainly reflected in three aspects: capillary force, surface chemical action, and physical barrier action.

Capillary force is the force produced by the phenomenon that liquid rises or falls in a thin tube, and this action is derived from the interaction between the surface tension of liquid and the wall of the thin tube [88]. In wood sponge, because of its porous structure with a three-dimensional network [62], these pores can be regarded as countless tubules. When crude oil comes into contact with the wood sponge, the crude oil molecules will be attracted by the surface tension of the pore wall [89], thus being sucked into the pores. The pore size is widely distributed, with samples ranging from micropores to macropores. The pore size distribution is beneficial to the effective adsorption of crude oil molecules with different viscosities. Moreover, the pores are interconnected, forming a complex network structure, which enables crude oil molecules to diffuse and transport freely in the pores [62]. When the crude oil comes into contact with the wood sponge, the crude oil will spontaneously penetrate into the pores due to the surface tension of the pore wall and the interaction of crude oil molecules. The infiltration process does not require external pressure or energy input. Moreover, the adsorption speed of crude oil under the action of capillary force is increased. The diffusion and transport of crude oil molecules in the pores are driven by the surface tension of the pore walls, thus realizing rapid adsorption.

The surface chemical action of wood sponge to adsorb crude oil is one of the keys to its high-efficiency adsorption performance. This effect is mainly based on the interaction between the surface of the wood sponge and crude oil molecules. In the preparation process of wood sponge, hydrophobic groups (such as polysiloxane [90], fluoride, etc.) are usually introduced onto its surface through chemical modification. These hydrophobic groups can significantly reduce the hydrophilicity of the surface of wood sponge [57], making it more inclined to interact with the oil phase (such as crude oil). The existence of hydrophobic groups causes the wood sponge to repel water molecules and preferentially adsorb crude oil molecules when it comes into contact with oily wastewater. There will be intermolecular interactions, such as the van der Waals force and hydrogen bonding, between the crude oil molecules and the hydrophobic groups on the surface of wood sponge [90]. These interaction forces enable crude oil molecules to be firmly adsorbed on the surface of the wood sponge. Therefore, the modified wood sponge can adsorb crude oil more effectively. In addition to the hydrophobic groups, the cellulose skeleton of wood sponge itself exhibits a certain degree of lipophilicity [91]. The lipophilicity enables the wood sponge to more effectively combine with crude oil molecules, thus improving the adsorption efficiency.

The pores in the wood sponge not only provide abundant space for the adsorption of crude oil, but also constitute a physical barrier [92], which can prevent other impurities in the water phase from entering the pores. The pores of wood sponge have certain pore size selectivity. This means that molecules of different sizes will be hindered to varying degrees when passing through the pores. The physical barrier enables the wood sponge to achieve efficient oil–water separation without adding any chemical reagents. Besides separating the oil–water mixture, the physical barrier function of the wood sponge can also prevent other pollutants (such as heavy metal ions and organic pollutants) in the water phase from entering the pores [93], thus protecting the adsorption performance and service life of the sponge.

## 3. Preparation Methods of Hydrophobic Wood Sponge

The first step in making hydrophobic wood sponge is to soak raw materials (usually balsa wood) in acid (such as acetic acid and sodium chlorite) or alkaline solution (such as sodium hydroxide and sodium sulfite), allowing them to react for several hours at a constant temperature in a water bath to remove most of the lignin and hemicellulose in the raw materials before cleaning them with deionized water and finally subjecting them to freeze drying to obtain an anisotropic compressible wood aerogel via a directional tube. Lignin and hemicellulose display physical and chemical connections and are easily hydrolyzed by acid, while lignocellulose exhibits relatively stable chemical properties and only physical connections with lignin and hemicellulose. Therefore, the lignocellulose skeleton can be retained after the removal of lignin and cellulose [94,95,96]. We introduce mainly two basic methods for preparing wood sponge: chemical vapor deposition and chemical liquid deposition; on the basis of the two methods, solar energy and light energy are used to improve the preparation technique.

### 3.1. Chemical Vapor Deposition Method

In order to make the wood sponge hydrophobic, chemical vapor deposition (CVD) technology, employing a silane coupling agent, is generally used to embed a superhydrophobic coating onto the surface of the skeleton, i.e., polysiloxane is formed on the surface due to silanization [57]. This method has been widely used in recent years. The preparation of hydrophobic wood sponge is roughly divided into three steps, as follows: (1) the process of preparing wood sponge by removing lignin and hemicellulose using acidic or alkaline solution, leaving only the cellulose skeleton in the wood; (2) the gelation process of placing the wood aerogel into a freezing chamber for drying, removing the water to prepare a porous aerogel; (3) the hydrophobic modification process of placing the modifier and the wood sponge into a dryer for silanization, removing the excess modifier using a room-temperature vacuum oven [72]. However, there are many wood channels displaying a low curvature in the wood sponge itself, providing the treated hydrophobic aerogel with the ability to transport anisotropic liquid. The obtained wood aerogel not only displays oleophilic and hydrophobic separation characteristics, but also shows excellent mechanical compressibility. For this method, Guan et al. [53] used methyltrimethoxysilane (MTMS), a cheap and commonly used silylating agent, via chemical vapor deposition.

It is worth noting that in the wood sponge production process, in addition to the use of silane as a modifier, many other compounds can be used for surface modification or functionalization to improve performance or provide new characteristics. These compounds can include low molecular weight reagents and polymers.

As a low molecular weight reagent, fluorosilane, which is similar to silane, is also often used to enhance the hydrophobicity of materials [97]. The introduction of fluorine atoms can significantly reduce the surface energy of materials, thus improving their waterproof and oil-resistant properties. Moreover, there are some organic acids, such as acetic acid and propionic acid [59,67], which can react with hydroxyl groups on the surface of wood to form ester bonds, thus changing the surface properties of the materials. However, it should be noted that this method may not be as effective as silylation or fluorosilication. In addition, some natural polymers, such as chitosan [98] and cellulose derivatives [99,100], offer good biocompatibility and biodegradability and can be used as coatings or modifiers for wood sponges. They can enhance the mechanical properties, hydrophilicity, or hydrophobicity of materials. Yi et al. [98] used chitosan to prepare wood aerogels for the efficient removal of oil from water. Wen et al. [101] used polyacrylic acid (PAA) as a modifier of wood sponge and introduced PAA into the wood cellulose skeleton using graft copolymerization, thus promoting good water absorption and water retention in the wood sponge. Li et al. [102] studied the surface modification of wood sponge using polyethylene glycol (PEG). The introduction of PEG not only improved the hydrophilicity of the material, but also helped to reduce its surface tension and promote the oil–water separation process. MTCS [59], PMHS [54], and TCMS reagents [103] are also commonly used in chemical vapor deposition.

After CVD treatment, the porosity of the wood is basically unchanged, and its hydrophobic and oil-absorbing ability is enhanced. At present, this method has been widely used to create aerogels for separating oil–water mixtures [94,95,96]. Although CVD can significantly improve the hydrophobicity of the substrate, in this method, the reaction conditions, including reaction temperature, pressure and time, and initial reagent dosage, must be accurately controlled. With the increase in sample size, the diffusion time and reaction time also increase. In addition, the internal grafting distribution of the materials treated using this method is often uneven, and the adhesion between the superhydrophobic surface coating and the substrate is weak, which also limits its application in the actual treatment of oily wastewater [57].

### 3.2. Chemical Liquid Deposition Method

The liquid deposition (LPD) method was originally employed as a unique process for preparing various metal oxide films via the wet method [57]. It was also discovered that this method can be used for wood-function integration, mainly by soaking wood sponge in evenly mixed aqueous solution to complete the reaction. This method has been gradually applied to the hydrophobic modification of wood sponge. Using balsa wood as the matrix, balsa wood blocks were first transformed into cellulose wood sponge. The hydrophobic wood sponge prepared by liquid-phase silylation was more hydrophobic than that prepared by CVD, and the internal hydrophobic angle could reach more than 130 degrees, which indicated that it possessed a stronger oil absorption capacity and better compression fatigue resistance. The hydrophobic wood modified using the LPD method also shows excellent superhydrophobicity under acidic, alkaline, and salt water conditions, as well as in hot water, which proves its excellent durability [104,105]. These results mainly result from the fact that, compared with CVD method, this process is very simple, and the silanization reaction on the cellulose skeleton is more comprehensive and uniform. In addition, the size and morphology of the object are not relevant, special equipment, such as a vacuum system, is not needed, and most of the reactions can be completed under mild conditions. More importantly, the hydrophobic treatment process does not require catalysts or toxic substances, which solves the problems of complex processes and poor sustainability encountered in the related research. Through experiments, it was proven that this method can yield a cellulose aerogel structure exhibiting robustness, mechanical compressibility, anisotropy, and superhydrophobicity, and it can then be used for reusable oil–water separation [106,107,108,109].

Lignocellulose is rich in hydroxyl groups, providing opportunities for chemical functionalization and hybridization with other components [110]. Under the conditions of humidity and mild heating, a small amount of water and ethanol dispersant are used to hydrolyze the silylating agent to form silanol. These silanols further react with hydroxyl groups or other silanols on the surface of wood sponge to form covalently connected silane layers, ultimately forming a cross-linked polymer [18,111,112].

Compared with CVD, the liquid deposition method can also be accurately quantified, and the optimal proportion can be determined by controlling the amount of silylating agent. Liu et al. [113] used a liquid phase hydrophobic modification strategy to realize hydrophobic modification. Firstly, the obtained wood sponge is reacted in situ in a water system, and the surface is modified using low surface energy materials (MTMS). The hydrophobic angle is the highest, reaching 159°, which exceeds that of the hydrophobic and oleophilic wood aerogels used in most cellulose-based materials [114,115,116]. When the amount of MTMS continues to increase, the contact angle will decrease slightly, which may be due to the formation of larger polysiloxane particles on the WS surface at this ratio, resulting in uneven surface roughness [117,118]. In silane treatment, the silane monomer or oligomer can easily diffuse into the wood cell wall in the solution, thus providing the functional structure with better performance [98]. In this way, the superhydrophobic wood sponge is more likely to lose its superhydrophobicity in a corrosive environment. The chemical stability of superhydrophobic surfaces [65,119] is a key feature for its practical application. This also shows that LPD, as a hydrophobic treatment method, is very reliable, uniform, and effective.

### 3.3. Improving the Preparation Method by Utilizing Solar Energy and Light Energy

In order to improve the oil absorption capacity of wood sponge, many researchers have tried different hydrophobic modifiers in their own research. In addition, the researchers accurately mastered the best reaction conditions for lignocellulosic gels prepared using the CVD or LPD method and the best preparation conditions for different modifiers by controlling the variables. Through an in-depth understanding of the oil absorption mechanism of hydrophobic sponge, many researchers have put forward a new method for oil–water separation, improving the sponge preparation method using solar and electric energy.

Because of the high viscosity of crude oil, the separation efficiency is seriously limited. However, the viscosity of crude oil decreases with the increase in temperature. Thus, many researchers suggest that solar and electric energy can be used to realize heat conversion on hydrophobic wood sponge, thus increasing the temperature of crude oil and enhancing the effect of oil–water separation [73,120,121]. In recent years, hydrophobic/lipophilic porous adsorbents, with excellent Joule and solar heating properties, have attracted increasing attention because of their high adsorption speed, high electrothermal conversion, and photothermal conversion efficiency [122,123,124,125,126]. The three-dimensional porous wood sponge, with low density, high porosity, and compressibility with this heat-absorbing coating, can be used as a self-heating absorber to soak up oil [123,127]. These coatings, commonly used in many commercial sponges, include dopamine coatings [128], reduced graphene oxide coating [71,129,130], carbon nanotube modification [131,132], the CuO@CuS package [133], etc. However, compared with the serrated pores of commercial sponges, the radially oriented microchannels of wood sponges not only improve the performance of the self-heating absorber, but also improve the oil absorption capacity through low transfer resistance [134,135].

For example, Chao et al. [73] used solar and electric energy to realize the thermal conversion of hydrophobic wood sponge, choosing to add graphene coating on wood sponge to improve its self-heating ability. Graphene can effectively capture photons in the lattice and then convert solar energy into heat energy through lattice vibration to increase the in situ fluidity of crude oil, showing high separation efficiency [120]. The researchers first obtained graphene oxide using the improved Hummers method [136]. Then, the obtained wood sponge was soaked in a graphene oxide (GO) dispersion, dried under vacuum for 10 h at room temperature, and freeze-dried. Then, samples were taken from a drying tray, sealed under vacuum, dried at 100 °C for 6 h to obtain the wood sponge, and the graphene coating was reduced in situ. Then, a layer of transparent octadecyltrichlorosilane (OTS) was deposited in n-hexane via simple dip coating to maintain the sample’s hydrophobicity and photothermal conversion ability [120].

Huang et al. [57] used electric energy to realize the thermal conversion of hydrophobic wood sponge. They developed a highly hydrophobic wood sponge coated with fluoroalkylsilane modified reduced graphene oxide (F-rGO@WS) for separating viscous crude oil from water. Firstly, chemical treatment was carried out to realize unobstructed longitudinal channels; then, a layer of graphene oxide was applied by simply immersing and coating the sponge, and then the sponge was reduced with ascorbic acid and grafted with perfluorooctyl triethoxysilane. The reduction of graphene oxide mainly endows WS with electrothermal ability [137]. The grafting of perfluorooctyl triethoxysilane aims at reducing the surface energy of reduced graphene oxide on the WS skeleton [73].

In addition, Zhang et al. [138] developed a polyurethane sponge coated with Ti_3_C_2_T_x_ MXene (Ti_3_C_2_T_x_@PU) to repair oil leakage; their results showed that the Ti_3_C_2_T_x_ layer exhibited good Joule heating and a significant photothermal effect. Wang et al. [73] used a new type of Ti_3_C_2_T_x_ MXene to wrap the wood sponge, and their results quickly cleaned up the crude oil leakage via excellent Joule heating and a good photothermal effect. Ti_3_C_2_T_x_ MXene is obtained by the selective etching of Ti_3_AlC_2_, as reported in the etching method [122]. First, 1 g of lithium fluoride and 20 mL of 9 M hydrochloric acid were mixed at room temperature. Secondly, 1 g of Ti_3_C_2_T_x_ powder was slowly poured into an HCl/LiF solution at 35 °C and allowed to react for 24 h, under continuous stirring. Thirdly, the obtained suspension was washed with ultrapure water several times until its pH value was greater than 5. Finally, the precipitate was ultrasonicated for 1 h and centrifuged to obtain Ti_3_C_2_T_x_ MXene nanosheets [121]. Ti_3_C_2_T_x_ MXene generates heat in situ, with the assistance of electricity and solar energy [139,140]. MXene comprises a large family of two-dimensional early transition metal carbides and/or carbonitrides [121], exhibiting high metal conductivity and a large specific surface area [141]. Li et al. [142] proved that the efficiency of Ti_3_C_2_T_x_ MXene in regards to thermal optic conversion is 100%.

The material used by the all of above researchers is fir, which is a natural renewable material with obvious low density and high mechanical properties [142]. Researchers can generate heat in situ through electricity and solar energy. Nanomaterials can be combined with hydrophobic wood sponge, which can effectively capitalize on the Joule-assisted and solar-assisted effect to generate heat in situ to efficiently clean and recover high-viscosity crude oil. This new type of wood sponge adsorbent, with high mechanical superelasticity and hydrophobicity/lipophilicity, displays outstanding advantages for crude oil leakage repair, and the use of Joule and photothermal heat can realize all-weather operation. Compared with ordinary sponge-like adsorption materials, wood sponge possesses a rare radial pore structure and excellent compressibility, which significantly improves adsorption rate and recyclability. The application of hydrophobic wood sponge can be divided into four categories: chemical vapor deposition, chemical liquid deposition, improving the absorption rate of crude oil by wood sponge using using solar heat, and achieving this improvement via electric energy.

**Figure 2:**
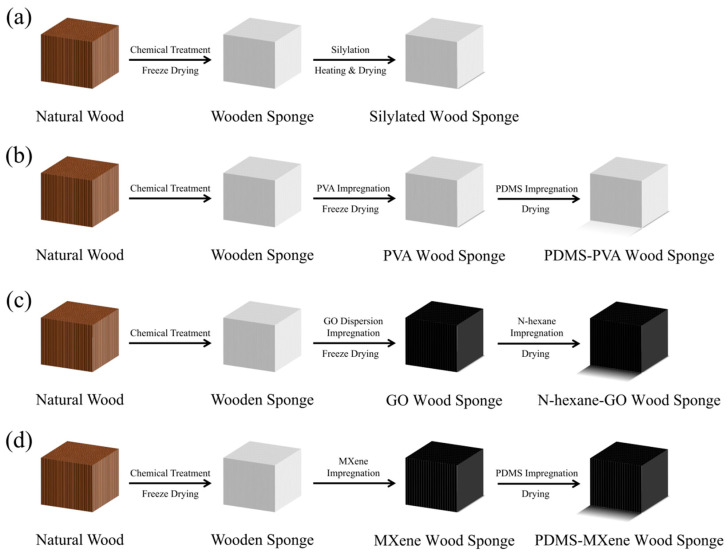
Development history of a hydrophobic wood sponge. (**a**) CVD, (**b**) LPD, (**c**) LPD and Joule heat collection using graphene nanocoatings; (**d**) LPD and added metal carbide nanosheet coatings utilize heat and electricity.

The change process, shown from top to bottom in the Figure 2, indicates that the research on the modification of hydrophobic wood sponge is gradually developing towards a more comprehensive and professional process. The preparation of wood sponge has gradually developed from a simple modification of hydrophobic and hydrophilic oil to the use of the thermal effect to accelerate the oil and water separation ability on the basis of the original high hydrophobic water, which also proved that this process resulted in the improved oil absorption ability of hydrophobic wood sponge. However, the results also show that hydrophobic wood sponges are becoming more expensive to make. Table 2 shows a comparison of the preparation methods of wood sponge.

## 4. Limitation of Wood Sponge Used in Oil–Water Separation

The wood sponge material shows both potential and advantages in the field of oil–water separation, but it also has some limitations. The following provides an analysis of the limitations of wood sponge for oil–water separation.

As a material itself, wood sponge exhibits some physical limitations. First of all, although wood sponge shows high oil absorption performance, its oil absorption capacity is still limited by the structure and preparation process of the material itself [146]. In practical application, a large number of wood sponges may be needed to achieve the ideal oil–water separation effect, which increases the cost and complexity of operation. In addition, the wood sponge material may be affected by physical wear, chemical corrosion, and other factors during use, resulting in its relatively poor durability [147]. This may affect its stability and reliability for long-term use. Moreover, due to the softness and elasticity of the wood sponge, it is easily affected by external forces and can be deformed during use [92], which may impact the effectiveness and accuracy of oil–water separation.

There are also some technological difficulties and challenges regarding the process of oil–water separation using wood sponge material. First of all, the oil–water mixture may contain various components and impurities, which may affect the oil absorption performance of the wood sponge [73]. For example, some chemicals may react with wood sponge, resulting in its performance degradation. Secondly, while pursuing high separation efficiency, we also need to consider the cost factor. The preparation cost, use cost, and subsequent treatment cost of wood sponge must be comprehensively considered. If the cost is too high, it may limit its popularization in practical applications [148]. In addition, the wood sponge will absorb a significant amount of oil and impurities in the process of oil–water separation. The accumulation of these oils, along with their residue and impurities, in wood sponge may cause difficulties during its regeneration and recovery [149]. Therefore, it is necessary to develop effective regeneration and recovery technologies to reduce treatment costs and decrease environmental pollution.

In order to resolve these limitations, further research and development work may be needed to optimize the design and performance of wood sponges specifically used for sewage treatment applications. This may include improving chemical treatment, freeze drying, and other preparation processes; introducing modifiers; and improving mechanical properties.

## 5. Summary and Prospect

Wood sponge is a type of wood aerogel with cellulose as the main skeleton; wood sponge is obtained by removing lignin and hemicellulose from natural wood. The surface energy of the sample can be changed via chemical modification, and sponges with different hydrophobic and oil-absorbing properties can be obtained [150,151,152]. There is a certain predictability between the preparation conditions and the properties of hydrophobic wood sponge, showing that its preparation process is controllable, and the most suitable preparation method can be identified by controlling the variables. After silylation modification, the adsorption capacity of hydrophobic wood sponge for oil and organic solvents is greatly improved, and this can also be recovered by simple extrusion. This recyclable green wood sponge exhibits high resilience, porosity, and oil absorption capacity, and the 3D aerogel material, displaying high porosity, light weight, and directional channels, is an attractive and promising candidate material for wastewater treatment. It is expected to be used in environmental protection fields, including sewage treatment and oil spill cleaning. At present, the research investigating the absorption of crude oil using wood sponge has achieved significant in-depth research results, reporting many breakthroughs regarding oil absorption capacity and reliability, e.g., Run et al. [21] proposed a superhydrophobic sponge with flame retardant properties that swells easily and can absorb 79–195 times its own weight in oil and organic solvents.

In the future, wood sponge preparation technology will tend to directly extract a biological matrix (such as chitosan, cellulose, chitin, and other polysaccharide structures) and then use directional freezing technology to achieve the special 3D structure, which is more suitable for large-scale production and application than are block-shaped wood materials. We anticipate that future research will focus on the practical application and commercialization of hydrophobic wood sponge materials, with an emphasis on strengthening their strong oil absorption, low cost, and high recyclability characteristics.

## Figures and Tables

**Table 1:** Results of oil–water separation using wood sponge and other materials.

Ref.	Material	Oil	Absorption Capacity	Porosity	WCA	Cycles of Reuse
[59]	Modified wood sponge	Cuclohexane	17,300 mg g^−1^	96.47%	121.37°	More than 10 times
[74]	Carbonaceous aerogel	Peanut oil	11,400 mg g^−1^	Volume of 4.13 g cm^−3^	144.2°	Not reported
[75]	Cabot thermal wrap	Dichloromethane	13,000 mg g^−1^	92.1%	127.6°	More than 10 times
[75]	Aspen Aerogels Spaceloft	Dichloromethane	16,000 mg g^−1^	86.1%	130.9°	More than 10 times
[76]	Popcorn-based carbon aerogel	Chloroethane	10,830 mg g^−1^	Volume of 0.095 g cm^−3^	151.6°	Not reported
[18]	Chitin sponge	Phenisin	58,000 mg g^−1^	Pore sizes of 20–50 μm	148.7°	Above 93% after 10 cycles
[39]	Spongy graphene	Toluene	86,000 mg g^−1^	Density of 12.5 mg cm^−3^	114.2°	Basically unchanged after 10 cycles
[77]	Cellulose aerogel	Ruby	18,400 mg g^−1^	97.3%	145°	Above 96.4% after 5 cycles
[78]	Polyurethane sponge	Chloroform	160,000 mg g^−1^	99.3%	127°	Basically unchanged after 50 cycles
[79]	Butyl rubber	Crude oil	23,000 mg g^−1^	Volume of 8.9 mL g^−1^	Not reported	Above 70% after 8 cycles
[79]	Polypropylene	Fuel oil	15,700 mg g^−1^	Pore sizes of 10 μm	Not reported	Above 10% after 8 cycles
[19]	Modified PU sponge	Lubricate oil	25,000 mg g^−1^	Not reported	Not reported	More than 300 times
[39]	Graphene sponge	Castor oil	75,000 mg g^−1^	Pore sizes of 570–620 μm	114.2°	More than 10 times
[80]	Graphene–CNT hybrid foam	Sesame oil	105,000 mg g^−1^	Pore sizes of 100 μm	152.3°	More than 6 times
[81]	CNT sponge	Mineral oil	126,000 mg g^−1^	98%	156°	Above 96% after 10 cycles
[82]	CNF/carbon foam	Wash oil	28,400 mg g^−1^	95%	140°	Not reported
[83]	Exfoliate graphite	Heavy oil	75,000 mg g^−1^	73–77%	Not reported	Not reported
[84]	Fir fibers	Grade-C oil	15,000 mg g^−1^	Not reported	Not reported	Above 78% after 8 cycles
[85]	EV/CNT	Diesel oil	26,700 mg g^−1^	Pore sizes of 5–10 nm	Not reported	Above 94% after 10 cycles
[81]	CNT sponges	Vegetable oil	130,100 mg g^−1^	Density of 5.8 mg cm^−3^	Not reported	More than 10 times
[81]	CNT sponges	Vegetable oil	32,300 mg g^−1^	Density of 25.5 mg cm^−3^	Not reported	More than 10 times
[86]	Corn stalk	Diesel oil	8600 mg g^−1^	Not reported	Not reported	Not reported
[87]	Kapok	Diesel oil	36,700 mg g^−1^	Pore sizes of 16.5 μm	102°	Basically unchanged after 15 cycles

**Table 2:** Comparison of preparation methods of wood sponges.

Ref.	Preparation Method	Wood Sponge Species	WCA	Absorption Capacity	Compression	Advantages	Disadvantages
[94]	CVD	Cellulose/graphene aerogel	153°	80–197	Compressible to 90%	Strong hydrophobic ability, diverse materials, and good mechanical properties	Uneven deposition and harsh reaction conditions
[143]		Silylated cellulose fibers	142°	51–142.9	Compressible in diesel oil		
[53]		Silylated wood sponge	151°	16–41	Compressible to 60%		
[17]		Silylated nanocellulose sponge	136°	49–102	Compressible to 96%		
[113]	LPD	Superhydrophobic wood sponge	159°	23–60	Compressible	Simple treatment process, mild reaction conditions, uniform deposition; durability and accurate quantification	Slow reaction speed and low product purity
[96]		Superhydrophobic microfibrillated cellulose aerogel	151.8°	116–260	Compressible		
[73]	Utilizing solar energy and light energy	Graphene–wood sponge	134.2°	7.28	Compressible to 90%	Rare radial pore structure; excellent compressibility, recyclability, and high adsorption rate	Complex technology, stability problems, and high cost
[144]		Methyltrichlorosilane treated PVA-CNF aerogel	150.2°	44–96	Compressible to 80%		
[145]		CNT sponge	156°	87–176	Compressible in ethanol		
[144]		PVA/cellulose nanofibril aerogels	Not reported	44–96	Compressible		

## Data Availability

Data availability is not applicable to this article, as no new data were created or analyzed in this study.
